# Heart rate monitored hypothermia and drowning in a 48-year-old man. survival without sequelae: a case report

**DOI:** 10.4076/1757-1626-2-6204

**Published:** 2009-08-18

**Authors:** Fredrik Koller Lund, Johan GR Torgersen, Hans Kristian Flaatten

**Affiliations:** Department of Anesthesia and Intensive Care Medicine, Haukeland University Hospital5021 BergenNorway

## Abstract

**Introduction:**

Victims of severe hypothermia and cardiac arrest may appear dead. They are often unresponsive to on-scene resuscitation including defibrillation while profoundly hypothermic. Several cases of extreme hypothermia and prolonged cardiac arrest with good outcome have been published. We present a case of heart rate monitored (by pulse-watch) hypothermia, prolonged cardiac arrest and survival with complete recovery of neurological functions.

**Case presentation:**

On December 22nd 2007 a physically fit, ethnic Norwegian 48-year-old male kayaker set out to paddle alone around an island in a Norwegian fjord. 3 hours 24 min into his trip the kayak capsized in 3.5°C seawater about 500m from the closest shore. The accident was not observed. He managed to call for help using his cellular phone. After a search and rescue operation he was found by our air ambulance helicopter floating, prone, head submerged, with cardiopulmonary arrest and profound hypothermia. He was wearing a personal heart rate monitor/pulse watch. Following extraction, he received cardiopulmonary resuscitation during transport by air ambulance helicopter to hospital. He was warmed on cardiopulmonary bypass from 20.6°C core temperature and return of spontaneous circulation was achieved 3h 27 m after cardiac arrest occurred. After 21 days of intensive care he was discharged from hospital 32 days after his accident. Testing revealed normal cognitive functions one year after the incident. He has returned to his job as an engineer, and has also taken up kayaking again. We provide heart rate and time data leading up to his cardiac arrest.

**Conclusion:**

Hypothermia has well established neuro-protective effects in cardiac arrest, as our case also shows. Simple cardiopulmonary resuscitation without use of drugs or defibrillation, should be continued until the patients can be re-warmed, preferably using cardiopulmonary bypass. This approach can be highly effective even in seemingly lost cases.

## Introduction

Severe hypothermia is commonly defined as core body temperature below 28°C. Cardiac arrest in hypothermia may occur as a pro-arrhythmic consequence of hypothermia itself, or asphyxia (e.g. drowning). Several cases of extreme hypothermia and prolonged cardiac arrest with good outcome have been published [1]. Individuals who maintain circulation to lower temperatures are more likely to survive neurologically intact after prolonged cardiac arrest [2].

Circulatory arrest causes hypoxemia and hypo-perfusion in cerebral tissue. The resulting cerebral hypoxia may cause brain damage due to anoxic cell death directly, or secondary to consequent cerebral oedema and resultant hypo-perfusion. The range in brain affection varies from complete anoxic brain damage to no permanent damage at all. Several factors can influence the degree of brain damage.

Falling body temperature slows metabolism decreasing oxygen consumption of human cells. This protects organs including the heart and central nervous system by delaying onset of ischemic injury.

## Case presentations

### Setting

A previously healthy and physically fit 48-year-old male kayaker of Norwegian ethnic origin sets out to paddle alone around an island (Varaldsøy) in the Norwegian Hardanger fjord - an estimated trip of about 4-5 hours.

### Outfit/equipment

Gore-Tex® shell with thin wool underneath. Flotation vest without collar. Waterproof GPS-receiver (Global Positioning System) and cellular phone in a waterproof container. Polar® heart rate monitor consisting of an ECG chest band and a wristwatch recording heart rate data.

## Meteorological conditions

Air temperature at the time of the accident: +6ºC.

Surface wind (major local variations due to terrain): Beaufort 5 (16-21kts).

Surface sea temperature +3.5ºC.

3 h 24 min into his trip the kayak capsized about 500 m from the closest shore.

The accident was not observed.

The kayaker has no memory of the accident, but presumably could not right his kayak while in it (i.e. Eskimo roll), exited and struggled to try to get back in. He used his cell-phone several times to call for help (data from his cell-phone log). After several failed attempts he managed to reach a friend. This was after 14 minutes in the rough, freezing waters and he could hardly talk. The friend heard splashing sounds and first thought the call was unintended. He was about to hang up when he heard the words: ”kayak” and ”Bondesundet” (a location in the fjord). With this limited information, he called the police. A SAR (search and rescue) operation commenced using a high speed local civilian vessel and our helicopter air-ambulance stationed in Bergen.

### Search

The helicopter was airborne 7 minutes after alarm, and arrived 20 minutes later over Bondesundet (22 NM from home base). The civilian high speed boat was already there searching. After 8 minutes search from the air the kayaker was found floating prone with his head completely submerged in seawater. There were no signs of life.

### Rescue

The kayaker was taken ashore in the high speed boat, and attempted single-helper CPR was started as it followed the helicopter to a nearby ferry dock. Once ashore quality CPR and endotracheal intubation was performed. Helicopter transport to our tertiary hospital was then undertaken while performing manual chest compressions. Ventilation was by an Oxylog® ventilator. No drugs were given, and defibrillation was not attempted. The only monitoring was capnography.

## Arrival at the hospital

### The initial treatment (Video 1)

The accident happened on a Saturday; hence the thoracic surgery team was summoned from home. Because of this, CPR by the pre-hospital team was continued into the cardiothoracic OR (Operating Room). On arrival the patient was asystolic with a bladder temperature of 20.6°C.

**Figure 1.**
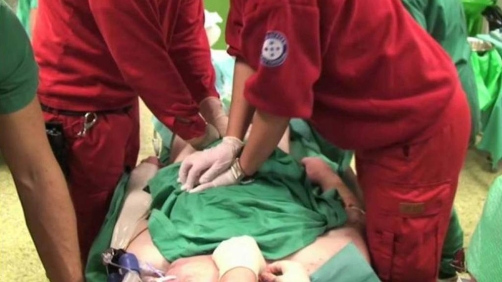
Interposed abdominal compression-CPR; Picture showing compression technique by two helpers. Frame captured from the video also included with this case report.

**Figure 2.**
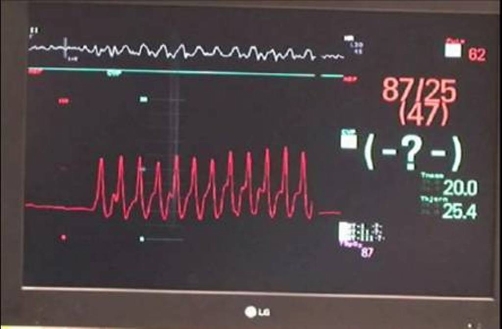
Arterial blood pressure during CPR; During surgery to establish femoral vascular access for the CPB, short coordinated pauses in chest compressions were needed. The picture shows the monitor shortly after such a break. Arterial pressure waves (red) from the restarted CPR are seen. The red numbers are the CPR induced arterial pressures systolic/diastolic (mean). The patient is asystolic or in fine ventricular fibrillation.

In the OR interposed abdominal compression**-**CPR [3] was applied. Monitoring invasive arterial blood pressure, this technique clearly produced higher arterial pressures than conventional chest compressions (Figure 1 and Figure 2).

### Preparing for re-warming on cardiopulmonary bypass

During surgery to establish femoral vascular access for the CPB, short coordinated pauses in chest compressions were needed (shown in video).

### Primary arterial blood analysis

pH 6,67, pO2 8,4 kPa, pCO2 17,0 kPa, BE -20 mmol/l, Hb 17,1 g/dl, Na^+^ 151 mmol/l, K^+^ 3,1 mmol/l, Cl^-^ 113 mmol/l, glucose 20,5 mmol/l and lactate 19 mmol/l.

### Cardiopulmonary bypass (CPB)

Full CPB circulation was established 2 hours after cardiac arrest occurred.

After core temperature reached 30ºC, the patient was in VF (ventricular fibrillation). Cardioversion was attempted 5 times, but VF returned until at 34°C, the VF became more course. A final shock was delivered and stable ROSC (Return of spontaneous circulation) was achieved.

### Treatment in the ICU

Following rewarming and ROSC the patient was intentionally kept hypothermic at 34°C the first 24 hours. Initially ha was treated for low cardiac output (cardiac index) < 2.0 l/m^2^ and pulmonary oedema. He was kept sedated and an ICP (intracranial pressure) transducer was placed to monitor for expected oedema-induced rise in ICP. A rise in ICP to 35 mmHg on day 3 led to treatment with Na-pentothal after which ICP fell to normal values. S-Na was intentionally kept around 150 mmol/l. Sedation was discontinued on day 5, and cardiac index had normalized at this time. He gradually regained consciousness the following days. Further complications included critical illness neuropathy, delirium and pneumonia. After 21 days of treatment in the ICU he was transferred to a conventional neurological ward. After a total of 33 days in hospital he was discharged to a rehabilitation unit.

## Data presentation and discussion

The data available in this case included the patient’s cell phone log, GPS data from the patient, GPS data from author^1^’s private hand-held GPS used in the helicopter during the search, the patients HRM/ pulse-watch and medical logs. A video was recorded by author^3^ during initial hospital treatment.

### SAR - GPS data

(From author’s private handheld PDA/GPS).

GPS based estimation of time of submersion/ drowning:

Assuming linear and constant speed movement of the objects in the water and by plotting lines through the points where we located the kayak and the kayaker at two times with known time-interval, we could estimate the time and position when and where these two objects parted in the water. This is likely the time and place of loss of consciousness. Cardiac arrest either caused this loss of consciousness of followed shortly thereafter. With an assumed error margin of +/-5minutes this yields 11-21 min submerged time before he was found.

This GPS based estimate correlates fairly well with the HRM data (21 minutes) which we used as the best estimate as GPS position data was not logged with great precision.

## The heart rate data

### Method

We used the software provided with the Polar® HRM for visualisation and analysis of his heart rate data. His telephone log and our air-ambulance log provided us with supplemental information.

**Figure 3.**
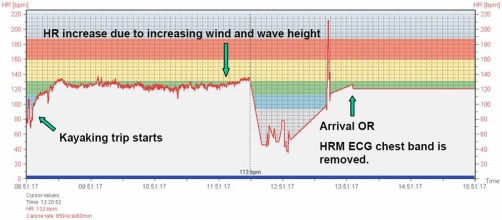
Heart rate data - The whole kayak trip; The graph shows the complete data of recorded heart rate from the start of the kayaking trip until data recording stops.

**Figure 4.**
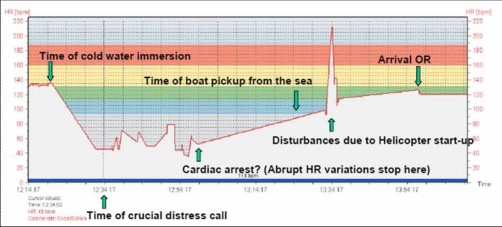
Heart rate data - Accident and rescue; A zoomed in version of figure 4. Heart rate data from just before the accident until arrival in the hospital operating room.

Note: The Polar® HRM is not intended for recording arrhythmias. Sadly, according to the manufacturer, a real-time ”error-correcting” algorithm in the device potentially changes abnormal data before it is stored. Thus asystole and very rapid ventricular rhythms are probably lost. Noise artefacts from extreme muscle activity may also disturb the recordings. The HR data is recorded and stored at 5 sec intervals. The data can be seen visualized in figures 3 and 4. Prolonged straight lines are assumed to indicate signal fault or no signal from the ECG electrodes. We have assumed there is real heart rate data with interpolated straight lines during periods of noise, missing or faulty data. Key times are indicated with green arrows and text (Figure 3 and Figure 4).

### Analysis of heart rate data

There is, as expected, a brief increase before a sharp drop in HR following cold water immersion. Heart rate first rises from approximately 130 min^-1^ to 137 min^-1^ and then drops to 43 min^-1^ after approximately 11 minutes. From there on the HR seems to vary between 78 min^-1^ and 35 min^-1^.

The variations in HR with repeated patterns of accelerations and decelerations are assumed to be caused by physical struggling e.g. trying to get back into the kayak.

38 minutes after immersion these variations stop, probably due to final incapacitation. Cardiac arrest and head submersion is likely to have occurred simultaneously or shortly thereafter. The next recorded HR is 35 minutes later at close to 100 min^-1^. Manual chest compressions at a rate of about 100 min^-1^ had commenced at this time and may be the reason for this recording. The following spike in HR to 210 min^-1^ coincides with helicopter start-up with patient onboard and may have been caused by disturbances from the helicopter.

It is of course possible but unlikely that all data after immersion is corrupt, but we have no way of knowing if this is the case. Completely straight lines are obvious errors, but we have assumed the points between these lines to be genuine data.

Disappointingly, the recorded heart rate data is clearly sporadic limiting further analysis (Table 1).

**Table 1.** Results

	Discharge ICU	3 months	0-3 months	12 months	3-12 months
MOT	-1.27	0.88		0.7	
∆ z-score			2.15		-0.18
DMS	-1.24	1.08		0.42	
∆ z-score			2.32		-0.66
SOC	-1.94	-1.49		-0.03	
∆ z-score			0.45		1.46

(The patient did not fulfil the PAL tasks and these results are excluded from the analysis).

**Table:** Key data

Total time of exposure in the sea:	64 minutes
Time in water before submersion/ cardiac arrest:	38 minutes
Time from cardiac arrest to professional CPR:	31 minutes
Total time of cardiac arrest before Cardiopulmonary Bypass:	121 minutes
First recorded cardiac rhythm (in OR before starting CPB):	Asystole
Core body temperature (bladder) on arrival OR:	20.6ºC
Total CPR time before CPB circulation established:	90 minutes
Running time CPB:	166 minutes
**Total time of cardiac arrest before sustained ROSC**	**207 min = 3 h27 m**

## Neurological, neuropsychological and functional outcome

### Alertness

On the time of discharge from ICU the patient was fully alert and achieved a Glasgow Coma Score of 15.

### Neurological examination

The neurological examination was conducted at the end of the ICU stay and revealed no signs of focal damage to the brain.

### Neurophysiological examination

EEG: Three months after discharge from the ICU the patient’s EEG showed non-epileptoform pattern without clinical significance.

### Motor neurography

Normal at three months.

### Sensory neurography

Reduced conduction velocity in fingers on both hands at three months.

### Electromusculography

Normal at three months.

### Neuropsychological examination

CANTAB (Cambridge Neuropsychological Test Automated Battery) [4] is a semi-automated neuropsychological test battery of memory, attention and executive functions using a touch screen “tablet” PC. Tests: motor screening test (MOT), short-term memory and forced decision-making (DMS), spatial planning and motor control (SOC), and visual memory and learning (PAL).

The patient’s results are compared to an integrated age- and sex-matched normal population giving us his results as a z-score. A z-score of 0 represents a result equivalent to the mean result in the normal population. [5]. A change in |z-score| > 1.0 indicates significant improvement or deterioration (Table 1).

## Discussion of results of cognitive testing

On discharge the patient’s motor (MOT), short-term memory (DMS) and spatial planning function (SOC) was significantly reduced compared to the reference group. He can be characterized as having a mild to moderate cognitive dysfunction. 3 months after discharge his performance on motor and short-term memory tests was above the average performance in the reference group and the improvement was significant. His performance on the spatial planning task was still significantly reduced and there was no significant change since discharge. 12 months after discharge from the ICU his performance was not significantly different from the reference group and his performance on the spatial planning task had improved significantly. The decline in performance on MOT and DMS at 12 months was not significant and is considered clinically irrelevant.

## Conclusion

Despite the dramatic accident and consequent critical illness our patient had documented cognitive function at, or above average compared to the normal population 12 months after discharge from the ICU. His increase in cognitive function was most prominent during the first three months after discharge. The patient has returned to his former job as an engineer and has taken up his kayaking again.

Hypothermia has well established neuro-protective effects in cardiac arrest, as our case supports. Drowning victims often swallow water on drowning and risk further aspiration of gastric content to the airways during resuscitation. Securing the airway preferably by endotracheal intubation is therefore very important. Other than this, performing quality basic cardiopulmonary resuscitation until CPB is established should be the mainstay of pre-hospital treatment. Preventing further decline in body temperature is generally also a good idea in hypothermia. This may not necessarily be the case in hypothermia with cardiac arrest during transportation to hospital for re-warming by CPB. Aggressive re-warming during such transportation may well be detrimental to cerebral outcome. As current European guidelines [6] suggest, we agree that defibrillation and use of epinephrine or other drugs have little place in the initial treatment of severe hypothermia with cardiac arrest.

We hope our case report can serve as inspiration to all health care providers presented with hypothermic cardiac arrest patients.

### Take home message

Cold cardiac arrest patients are not dead until both warm and dead.

## Supplementary File

Initial treatment Rewarming by cardiopulmonary bypass. Video showing initial hospital treatment including interposed abdominal compression CPR, connection to CPB and successful defibrillation to ROSC. Also included is a short clip from the press-conference with the patient after his incident.http://casesjournal.com/casesjournal/article/downloadSuppFile/6204/19445

## Abbreviations

CANTAB: Cambridge Neuropsychological Test Automated Battery
DMS: short-term memory and forced decision-making
GPS-receiver: Global Positioning System
HRM: Heart rate monitor
ICP: intracranial pressure
MST: motor screening test
OR: Operating Room
PAL: visual memory and learning
ROSC: Return of spontaneous circulation
SAR: search and rescue
SOC: spatial planning and motor control
VF: ventricular fibrillation
